# A graphical model approach for inferring large-scale networks integrating gene expression and genetic polymorphism

**DOI:** 10.1186/1752-0509-3-55

**Published:** 2009-05-27

**Authors:** Jen-hwa Chu, Scott T Weiss, Vincent J Carey, Benjamin A Raby

**Affiliations:** 1 Channing Laboratory, Brigham and Women's Hospital, Harvard Medical School, Boston, MA 02115, USA; 2 Division of Pulmonary and Critical Care Medicine, Brigham and Women's Hospital, Boston, MA 02115, USA; 3 Center for Genomic Medicine, Brigham and Women's Hospital, Boston, MA 02115, USA

## Abstract

**Background:**

Graphical models (e.g., Bayesian networks) have been used frequently to describe complex interaction patterns and dependent structures among genes and other phenotypes. Estimation of such networks has been a challenging problem when the genes considered greatly outnumber the samples, and the situation is exacerbated when one wishes to consider the impact of polymorphisms (SNPs) in genes.

**Results:**

Here we describe a multistep approach to infer a gene-SNP network from gene expression and genotyped SNP data. Our approach is based on 1) construction of a graphical Gaussian model (GGM) based on small sample estimation of partial correlation and false-discovery rate multiple testing; 2) extraction of a subnetwork of genes directly linked to a target candidate gene of interest; 3) identification of cis-acting regulatory variants for the genes composing the subnetwork; and 4) evaluating the identified cis-acting variants for trans-acting regulatory effects of the target candidate gene. This approach identifies significant gene-gene and gene-SNP associations not solely on the basis of gene co-expression but rather through whole-network modeling. We demonstrate the method by building two complex gene-SNP networks around Interferon Receptor 12B2 (IL12RB2) and Interleukin 1B (IL1B), two biologic candidates in asthma pathogenesis, using 534,290 genotyped variants and gene expression data on 22,177 genes from total RNA derived from peripheral blood CD4+ lymphocytes from 154 asthmatics.

**Conclusion:**

Our results suggest that graphical models based on integrative genomic data are computationally efficient, work well with small samples, and can describe complex interactions among genes and polymorphisms that could not be identified by pair-wise association testing.

## Background

Jansen and Nap [1] proposed expression quantitative trait locus (eQTL) mapping by considering gene transcript abundances as quantitative phenotypes. Identified eQTLs could then be tested as potential disease-susceptibility candidates in genetic association studies, with the expectation that variants with functional influence on gene expression would have a higher likelihood of influencing clinical traits. Initial studies examining the feasibility of such integrative genomic strategies in a variety of model organisms and in human populations have demonstrated that a substantial proportion of transcripts exhibit heritable expression, and initial genome-wide eQTL surveys have identified putative regulatory variants for several thousand genes [2,3]. Most of the regulatory variants identified to date are situated proximal to the target transcript (i.e. cis-acting variants) despite the much larger search space for distal variants (i.e., those in trans), and it is notable that regulatory variation has been identified for only a fraction (~13.1 14.8% in [2] and [3], respectively) of the transcripts demonstrating heritable expression. It is likely that much of the remaining unexplained heritable variation is due to unidentified trans-acting or epistatic effects. However, identifying these effects has become an extremely difficult problem, with the number of potential tests dwarfing the number of samples available for analysis. One potential solution is graphical modeling. Graphical modeling is a powerful tool for describing complex interaction patterns among variables in high-dimensional data used frequently in microarray analysis [4]. Though graphical modeling provides a simple way to infer and visualize complicated networks among genes and gene products, estimating such networks can be very difficult when considering the impact of millions of variant-gene combinations. To overcome this problem, Schäfer and Strimmer [5] proposed an empirical Bayes methods for fitting Gaussian graphical models, which performs well in inferring large-p small-n gene networks. In this paper, we extend their method and develop new strategies to infer a gene-SNP network in an integrative genomic setting. First we build the gene network based on the empirical Bayes method, then we identify a subnetwork of genes (edges) that interact with a candidate gene of interest (target gene). Finally, we identify cis-associated variants (arbitrarily defined as SNPs located within 50 Kb of the genes) for the interacting genes to complete the network and identify those cis-acting variants that in turn have trans-acting effects (greater than 50 Kb) on the target candidate gene. The genetic association testing is conditional on the developed graph network; the method is robust to false-positive association due to collinearity. Herein, we describe the basic model and the methods for its inference, its validity (through computer simulations), and its application to several data sets.

## Methods

**Figure 1:**
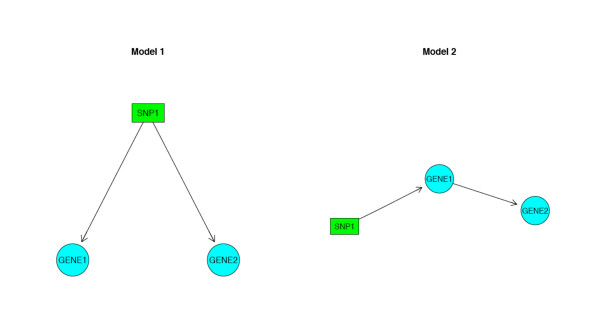
**Two types of SNP-gene modules**.

**Figure 2:**
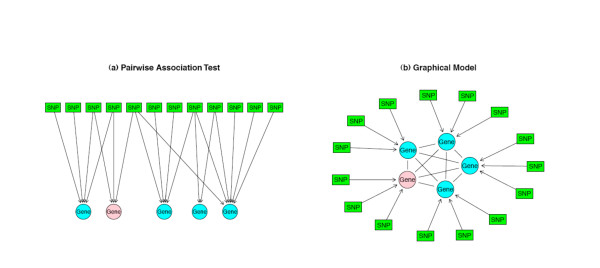
**(a)Association test vs. (b)graphical models**. Pink node denotes the candidate gene.

To demonstrate the key features of our model, consider a very simple network consisting of one SNP and two genes, where the measured transcript abundance of each gene is significantly associated with SNP genotype. Figure 1 shows two possible modules that could explain the observed associations. These modules are analogous to the Independent (Model 1) and Causal (Model 2) models in [6], respectively. In Model 1 the SNP influences the two genes independently, while in Model 2 the SNP influences gene 2 through gene 1. We note that pair-wise association testing cannot distinguish between the two models. Figure 2 contrasts more complex, genome-wide association testing. Figure 2a depicts genome-wide pair-wise association testing, akin to that from recent studies of the genetics of gene expression [2,3], whereas Figure 2b depicts the use of graphical modeling to test for SNP-gene associations while considering the interdependence of co-expressed genes.

### Model

Here we describe the GGM of the gene-SNP network. Our observed data include a gene matrix *Y *with *G *genes and *N *samples, and a SNP matrix *Z *with *H *SNPs and *N *samples. The model for the gene matrix follows [5], where *Y *follows a multivariate normal distribution:



where *y*_*gi *_represents the expression observation for *g*th gene in the *i*th sample, *μ *is the mean vector and Σ is the covariance matrix.

Let *H*_*g *_denote the collection of SNPs within 50 Kb upstream and downstream of the transcription start cite of gene *g*, *g *= 1, ... *G*. Similarly, define *G*_*h *_as the collection of genes in the 50 Kb neighborhood of SNP *h*, *h *= 1, ... *H*. *K*_*g *_and *K*_*h *_represent the number of variables in *H*_*g *_and *G*_*h*_, respectively.

We assume that there is an unobserved continuous variable *W*_*h *_driving the SNP *h *and *Y *and *W *follow a joint normal distribution:



where the matrix *V *represents the association between genes and SNPs and we assume that *V*_*gh *_= 0 if *g *∉ *H*_*g *_and *h *∉ *G*_*h*_.

The observed SNP matrix *Z*, represented in minor allele count and take value of 0,1, or 2, is related to *W *in the following way:



To enable development of a computationally feasible algorithm, our model includes several reasonable assumptions:

1. Multivariate normality: We assume that gene expression and SNP genotypes follow a multivariate normal distribution. Multivariate normality may be a good approximation for gene expression given the preprocessing procedures for expression measurements. Though SNP genotypes are discrete, normality is assumed to drive genotype effects under an additive genetic model.

2. Linear or pair-wise association: We assume that associations between the gene and polymorphisms are linear and there are no higher-order interactions. We recognize that higher-order relationships are likely present, but modeling such relationships, especially with a small sample, can be a very difficult problem.

3. Conditional independence of SNPs: We assume a unidirectional causal relationship between a SNP and a gene (that is, variants can alter gene expression whereas gene expression does not alter genoytpe). Given this assumption, the graphical model theory tells us that, if two or more SNPs are highly correlated (that is, in high linkage disequilibrium, LD), their effects on gene expression are statistically indistinguishable in our model. Therefore, although LD is typically present in genome-wide SNP datasets, we can (incorrectly) assume that all SNPs are independent conditional on all genes.

### Estimation

As the genes and SNPs considered in the model usually outnumber the samples, we take a multistep approach that takes advantage of the conditional independence assumption to estimate the gene-SNP network based on the correlation *σ*_*Y *_and *V*:

1. First estimate the covariance Σ_*Y *_for *Y *based on the shrinkage estimation described in [7]. The partial correlation matrix ∏ can be derived from the inverse of Σ_*Y*_. The standard graphical model theory shows that gene *i *and gene *j *are independent if the corresponding partial correlation coefficient ∏_*ij *_is zero. Schäfer and Strimmer [5] describe a testing procedure for the null hypothesis ∏_*ij *_= 0. Alternatively, we can calculate the empirical posterior probability that ∏_*ij *_≠ 0, i.e., there is an edge between *i *and *j *in the graph.

2. Even though it is possible to analyze the whole gene network in the next steps, sometimes one wishes to focus on part of the gene network and the corresponding cis-acting SNPs. For example, we might be interested in a particular candidate gene and the network surrounding it. It is straight forward to extract the subnetwork that we need, as the subnetwork can be defined as the collections of edges connected to the candidate genes that passed a certain threshold of FDR (False Discovery Rate) adjusted p-values or posterior probabilities *P*(∏_*ij *_≠ 0|*Y*).

3. Let G* be the collection of genes in the subnetwork that we are interested in; we can narrow down the SNP list to a subset H*:



For each *h *∈ H*, to avoid testing against all genes we consider the collection , which is the union of *G*_*h *_and the genes connected to a member of *G*_*h*_, we use the observed SNP data *Z*_*h *_as an approximation of the underlying variable *W*_*h*_:



where  and  represent the gene data and covariance matrix for , respectively. Now the partial correlation coefficient for SNP *h *related to *V*_*h *_and  can be estimated in the same way as in step 1. Notice that the partial correlations among members in  have been established on the basis of the whole-gene matrix *Y*, so we can ignore the result from  here.

4. The same FDR multiple testing or posterior probability thresholding procedure as in step 2 can be applied to the partial correlation coefficients between genes and SNPs. After we identify the significant edges the network is complete.

### Simulation Studies

To assess the sensitivity and specificity of the GGM approach, we performed a series of simulation studies. We generate a partial correlation matrix from which our gene expression data matrix will be generated. Generating a random correlation matrix that is both realistic and contains strong correlations is a challenging problem as it is difficult to assign random correlation coefficients and make it a positive definite matrix. Schäfer and Strimmer [5] use an algorithm generating random diagonally dominant matrices, which often produces very weak associations and structures unlikely to resemble real gene networks. As a result, the power to detect those weak links can be very poor. To simulate more realistic networks, we used the CAMP integrative genomics dataset. We fit a GGM for the 3,191 most variably expressed genes, from which we derive the 100 genes with highest correlation. We preserve all the significant correlation coefficients (posterior probability ≥ 0.5)and set the remaining non-significant correlations to zero. The resulting simulated true gene network includes 256 significant edges out of 4,950 possible edges. Once this partial correlation matrix is established, it is straightforward to derive the covariance matrix Σ_*Y *_and sample *Y *from it.

The locations for 100 genes and 2,500 SNPs are sampled from uniform (0,1) distribution. Cis-acting subsets *G*_*h *_and *H*_*g *_are defined as genes and SNPs within 0.005 of each other. The partial correlation coefficient between each SNP *h *and genes in *G*_*h *_follows a mixed distribution of 50% probability zero and 50% probability from uniform (-1, 1) distribution. Once the partial correlation coefficients have been assigned, they can be combined with , the rows and columns corresponding to *G*_*h *_extracted from the master correlation matrix ∏, to form a joint correlation matrix for SNP *h *and *G*_*h*_. The matrix can be made diagonally dominant and standardized, following the algorithm by [5], then converted to the joint covariance matrix . To generate the SNP data, first we generated *W*_*h *_from its conditional distribution:



After *W*_*h *_has been generated, we will assign cutoff points *a*_0 _and *a*_1 _to convert it to minor allele count. The cutoff points are determined by a random sample from 534,290 genotyped SNPs and make the distribution of the genotypes identical to the real data.

## Results and Discussion

### Simulation Results

The performance of GGM was evaluated with both large (N = 200) and small (N = 50) datasets. The simulated gene network contains 256 true edges. With large-sample data GGM identified 140 significant edges (54.69%), out of which 126 are true (90%), while only 97 (37.89%) significant edges (83 of which are true, 85.57%) were identified with small-sample data. The false-positive rate is about 0.3% in both cases. In [7] the power (the proportion of true edges identified) is about 50–60% for N = 200 and about 10–20% for N = 50; therefore our observed power is comparable to the simulation results in the large-sample case, and significantly better in the small-sample case, which may be due to the design of the "true network" based on a real data set.

We next added SNP data to the defined networks by testing the association between the SNPs and the genes for gene pairs with strong links. The large-sample case simulation includes 1,777 true cis-acting (i.e., within 0.005) SNP-gene associations. Though conditional dependency between SNPs and genes mapping to distances greater than 0.005 were permitted in the simulation (that is, cis-associated SNP with gene A could be associated with gene B due to correlations between genes A and B), we did not simulate any independent trans-acting effects. We detected 1,120 significant associations (63.03%), of which 1,113 were true (99.38%, i.e., only 7 false positives). We note that no significant independent trans-acting associations were detected, consistent with the simulated model. In contrast, failure to consider conditional dependence would reveal a large number of indirect associations: linear regression of all possible SNP-trait associations (regardless of distance) identifies 507 significant associations (out of a possible 3,447), though these associations are all indirect. As expected in light of the large number of comparisons performed, SNP-association mapping in the small-sample case is underpowered (only 170 of 1,244 true associations are detected, 13.67%), though no false-positive cis-acting association were observed, and again, no false-positive direct trans-acting associations were identified (compared with 61 of 2,414 detected by linear regression).

### Asthma Integrative Genomics Dataset

An integrative genomics study of the genetics of gene expression was performed in a subset of young adult asthmatics (n = 299) participating in the Childhood Asthma Management Program (CAMP) Phase 2, a multicenter follow-up study of childhood asthma. CD4+ lymphocytes were isolated from peripheral blood samples with anti-CD4+ microbeads by column separation (Miltenyi Biotec, Auburn CA). Total RNA was extracted from CD4+ lymphocytes with the QIAGEN RNeasy Mini Protocol (Valencia, CA). Expression profiles were generated with Illumina HumanRef8 v2 BeadChip oligonucleotide arrays (Illumina, San Diego CA) and scanned with the BeadArray scanner. Raw expression intensities were processed with the lumi package [8]. Each array underwent background adjustment with RMA convolution [9] and log2 transformation for variance stabilization. The combined samples were quantile normalized. Adequate DNA for genome-wide genotyping was available for 154 subjects of self-reported white ancestry with RNA samples. Genotyping was performed with the Illumina Infinium II HumanHap550 Genotyping BeadChip. We apply our method to a dataset of genome-wide SNP genotype data (534,290 autosomal markers) and peripheral blood CD4+ lymphocyte gene expression profiles (22,177 transcripts) in 154 asthmatic subjects. Schäfer and Strimmer [7] suggest that the power and positive discovery rate drop to zero when the number of genes outnumbers the samples by fivefold. These effects are also evident in our simulations. We therefore performed necessary data reduction using genefilter [10] by focusing only on 3,203 RefSeq-annotated genes variably expressed across samples (minimum interquantile range of 1.0 on log2 scale). Though p exceeded n by more than 20-fold, our GGM modeling identified 513,203 gene-gene associations (i.e., edges) with posterior probability ≥ .50, representing 10.01% of all possible  edges. To validate the gene network, we apply the same algorithm on an independent, publicly available gene expression set from CD4+ lymphocytes in both asthmatics and normal subjects (GEO series 473, see [11]). With a more stringent threshold (posterior probability ≥ 0.90), we find that a significant proportion of edges overlap in the two data sets (1,913 edges, *p *= 5.5 × 10^-106^). We also find 40 genes with over 100 significant connections with other genes in both datasets [See Additional file 1], which can be considered as hubs [12]. The extensive and reproducible connectivity of these 40 hub genes suggests they are of particular biological interest in CD4+ lymphocytes.

To illustrate the utility of network modeling using GGM, we focus on the network segment surrounding one of the identified hub genes – the beta-2 subunit of the Interleukin-12 receptor (IL12RB2, OMIM *601642). The IL12 receptor is constitutively expressed on CD4+ lymphocytes and is induced by antigen receptor triggering, and its ligand (IL12) is a potent immunomodulator in allergic airways disease [13]. GGM identified 306 genes with direct edges to IL12RB2 in the CAMP dataset [see Additional file 2]. 5,611 SNPs map to within 50 kb of these genes. Because the number of SNPs per gene is generally small (18.34 SNPs on average), we can efficiently estimate partial networks over the 5,611 SNPs quickly, as described in step 3. After FDR adjustment we identify 225 SNP-gene pairs (4.01%) with significant association [see Additional file 3]. Given the simulation results, we expect few of them to be false positives. Target candidates of interest include RAP1A [14] and TBKBP1 [15].

SNP-association mapping is productive for non-hub genes as well. For illustrative purposes, we mapped regulatory variants for genes linked to Interleukin-1B (IL1B, OMIM *147720), another biological asthma candidate gene. 353 SNP-gene pairs (5.2% of all cis-acting pairs tested) with significant association were identified with FDR-adjusted p-value ≤ 0.05 [see Additional file 4].

### GeneVar Dataset

We used the publicly available GeneVar dataset  to assess the reproducibility of our network building. GeneVar consists of gene expression profiling of Epstein-Barr virus-transformed lymphoblastoid cell lines for the 270 HapMap Consortium (Phase II) individuals and their available genotype data (3,967,792 SNPs) [3].

We apply our method to the 30 Caucasian trios in GeneVar. The dataset contains expression values from 47,293 probes for each individual, and we use the same filtering criteria as in CAMP to filter it down to 2,247 genes. The gene network contains 146,310 (5.8%) significant edges with posterior probability greater than 0.5, 67,345 (2.67%) with posterior probability greater than 0.8, and 44,207 (1.75%) greater than 0.9. With only 90 samples in the dataset, the latter categories are likely most reliable.

**Figure 3:**
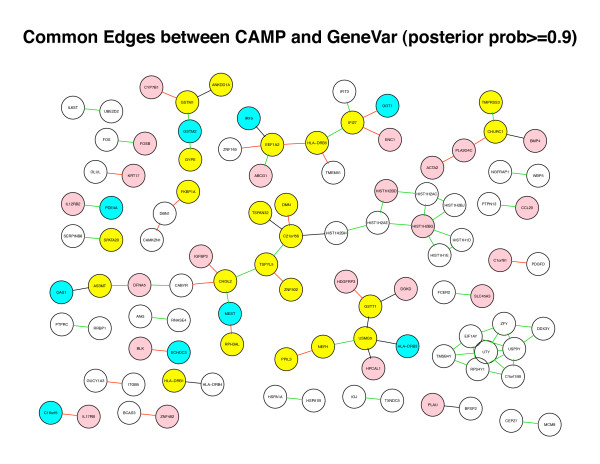
**Sub-network of 86 edges appeared in both CAMP and GeneVar network using a subset of 608 genes and threshold of posterior probability of 0.9**. Color nodes denote hubs (pink = CAMP, cyan = GeneVar, yellow = both). The color of the edges denote the direction (Green = both positive, Black = both negative, Red = opposite).

To compare the networks from GeneVar and CAMP, we focus on a subset of 608 genes that appear in both datasets after filtering. Using the posterior probability threshold of 0.9, we find 3,302 significant edges in CAMP and 2,239 in GeneVar. There are 86 edges that appear in both sets (See Figure 3), considerably more than would be expected by chance (p-value of 6.77 × 10^-11^), but far fewer than the large overlap of 1,913 noted between the two datasets.

IL1B is in both datasets after filtering. Using the 0.9 threshold, we find 51 genes directly linked to IL1B in GeneVar, and we estimate the gene-SNP network with 5,404 SNPs within 50 Kb of those genes. We find 133 significant SNP-gene pairs [see Additional file 5]. In CAMP, however, there are only 4 genes connected to IL1B in the reduced dataset and no overlap with GeneVar. Note that the subset accounts for only 2.74% of the genes in the original dataset.

We note that graph models can facilitate biologic interpretation of observed associations of SNP with multiple genes by distinguishing between conditionally independent (Model 1 in Figure 1) and dependent (Model 2) SNP-gene associations. For example, of the 133 significant SNP-gene associations noted in the GeneVar IL1B subset analysis, 25 (18.8%) of these SNPs are associated with IL1B expression in a univariate analysis (for example, by linear regression of SNP genotype on the expression trait – see Additional file 5). None of these trans-associations remain significant when the effects of the cis-associated genes are considered, suggesting that the SNPs identified influence IL1B expression by first altering the expression of the cis-associated gene, thereby strengthening support for a true gene-gene interaction. Without considering conditional dependency, these insights would be missed.

## Conclusion

Identifying regulatory sequence variants that impact gene expression will be helpful for the identification of functional disease susceptibility genes. Though several published integrative genomic studies have highlighted the utility of these approaches, the current bipartite-univariate approach (see figure 2a) limits the scope in which the problem can be explored. As we see in [12] and [16], the underlying biology network between genes and other phenotypes often cannot be adequately described by a bipartite graph. In this paper we describe a novel approach using a GGM to infer a gene-SNP network based on the algorithm by [5]. Our results from simulations and applications show the method works well in large-*p *small-*n *settings. The simulation results are similar to [17] in terms of sensitivity and specificity, which compare favorably with other standard methods in large-*p *small-*n *settings, such as LASSO [18]. We have also found that the networks derived with GGM are fairly reproducible across real datasets, as manifest by the significant overlap between the CAMP CD4+ lymphocyte data and other datasets. The graph networks presented here are intuitive and easy to visualize. We also note that once we identify the candidate genes and the set of cis-acting SNPs to test on, the algorithm for superimposing SNP associations can be run in minutes on a desktop computer.

One natural extension of this method is to integrate additional clinical phenotypes (such as disease status or quantitative traits) with the genotype and expression data network and explore the interaction among genes, regulatory variation, and clinical phenotype to enable identification of critical disease susceptibility loci. We recognize that the prohibitively high dimensionality of the data will still pose a problem (particularly if sample size is small), and, as we rely on the assumption of conditional independence in this paper, we may need additional assumptions in the disease network for a computationally feasible solution.

## Authors' contributions

The statistical model and methodology were developed by JC based on the concept by BAR with the support of VJC for statistics and bioinformatics. STW is the PI for CAMP project and the experimental data were provided by BAR. The manuscript was written by JC and all co-authors have approved the final version.

## Supplementary Material

Additional file 1 **List of 40 genes with 100 or more direct edges in both CAMP and GEO473**. This table includes 40 genes with over 100 significant connections with other genes in both CAMP and GEP473 datasets. These genes can be considered as "hubs". Click here for file

Additional file 2 **List of 306 genes with direct edges with IL12RB2 in CAMP**. This table includes 306 genes with a significant direct connection with IL12RB2 in the gene-gene network in the CAMP dataset. Click here for file

Additional file 3 **List of 225 significant gene-SNP associations in CAMP connected to IL12RB2**. This table includes 225 significantly associated gene-SNP pairs where the gene is connected to IL12RB2 in the gene-gene network in CAMP dataset. Click here for file

Additional file 4 **List of 353 significant gene-SNP associations in CAMP connected to IL1B**. This table includes 353 significantly associated gene-SNP pairs where the gene is connected to IL1B in the gene-gene network in CAMP dataset. Click here for file

Additional file 5 **List of 133 significant gene-SNP associations in GeneVar connected to IL1B**. This table includes 133 significantly associated gene-SNP pairs where the gene is connected to IL1B in the gene-gene network in GeneVar dataset, including 25 SNPs which are associated with IL1B in the univariate analysis. Click here for file
