# Supplementary material for: A graphical model approach for inferring large-scale networks integrating gene expression and genetic polymorphism
Source: BMC Syst Biol. 2009 May 27;3:55. doi: 10.1186/1752-0509-3-55 (PMC2694152; doi:10.1186/1752-0509-3-55)
Supplement: Additional file 1 — List of 40 genes with 100 or more direct edges in both CAMP and GEO473. This table includes 40 genes with over 100 significant connections with other genes in both CAMP and GEP473 datasets. These genes can be considered as "hubs". [file 1752-0509-3-55-S1.pdf]

Table 1: List of 40 genes with 100 or more direct edges in both CAMP and GEO473

Gene	CAMP connection frequency	GEO473 connection frequency
DPYS	104	101
DRD3	282	109
DSC1	251	117
FBLN2	132	194
HOXD3	137	124
IL12RB2	213	108
PCTK3	139	100
PTPRN2	147	117
SLC13A1	205	112
TNNT1	280	127
ZNF135	230	126
ZNF155	130	100
TCAP	142	105
MYOM2	422	123
FADS2	143	100
CACNG2	137	106
FST	331	105
UTS2	380	109
SOX30	128	106
CAPN11	397	114
ABCB9	191	116
CNTNAP2	297	120
OPLAH	129	103
TRIM17	183	120
QPCTL	103	106
ZSCAN2	138	101
TMEM51	109	107
ZNF532	197	124
DOK4	131	105
LRP2BP	187	125
KIAA1967	127	118
DPEP3	165	106
MMP28	113	102
FSD1	233	134
THAP9	128	119
APOLD1	278	110
MFSD7	140	114
NAV1	156	109
TBC1D16	166	166
PTRF	248	103
