# Supplementary material for: A graphical model approach for inferring large-scale networks integrating gene expression and genetic polymorphism
Source: BMC Syst Biol. 2009 May 27;3:55. doi: 10.1186/1752-0509-3-55 (PMC2694152; doi:10.1186/1752-0509-3-55)
Supplement: Additional file 2 — List of 306 genes with direct edges with IL12RB2 in CAMP. This table includes 306 genes with a significant direct connection with IL12RB2 in the gene-gene network in the CAMP dataset. [file 1752-0509-3-55-S2.pdf]

Table 2: List of 306 genes with direct edges with IL12RB2 in CAMP

Gene	partial correlation	p-value	adjusted p-value	posterior probability
RPH3AL	0.040928632	2.22E-16	2.07E-13	1
QRSL1	0.036826263	2.22E-16	2.07E-13	1
TMPRSS13	-0.036581806	2.22E-16	2.07E-13	1
DPYSL4	-0.036503931	2.22E-16	2.07E-13	1
IL18RAP	0.03562025	2.22E-16	2.07E-13	1
SLC27A2	0.034069289	2.22E-16	2.07E-13	1
NEFL	-0.033956011	2.22E-16	2.07E-13	1
DBH	-0.032689389	2.22E-16	2.07E-13	1
CYP4F12	-0.030199698	5.11E-15	3.77E-12	1
CXorf6	0.030186523	5.33E-15	3.92E-12	1
ADARB2	-0.030043102	7.11E-15	5.12E-12	1
PRSS16	0.02916633	4.17E-14	2.59E-11	1
LOC399900	-0.029002206	5.77E-14	3.47E-11	1
CXCR6	0.028977725	6.06E-14	3.63E-11	1
FAM19A1	-0.027788285	6.09E-13	2.94E-10	1
FLJ10490	0.026855558	3.48E-12	1.41E-09	0.99999999
HOXB4	0.02672024	4.46E-12	1.76E-09	0.99999998
DSCAML1	-0.026623532	5.33E-12	2.07E-09	0.99999998
RNF175	-0.026584514	5.72E-12	2.20E-09	0.99999998
FLJ39501	0.026520243	6.43E-12	2.45E-09	0.99999998
SLC25A29	-0.02630007	9.57E-12	3.49E-09	0.99999997
WWC1	-0.026099295	1.37E-11	4.81E-09	0.99999996
ARL17P1	-0.026028981	1.56E-11	5.39E-09	0.99999995
TMCC1	-0.025680871	2.89E-11	9.38E-09	0.99999992
IGSF4	0.025651902	3.04E-11	9.81E-09	0.99999992
POLR2J2	-0.025635292	3.13E-11	1.01E-08	0.9999999
PCSK1N	-0.025591053	3.38E-11	1.08E-08	0.99999989
UTS2	0.025502574	3.95E-11	1.24E-08	0.99999988
TMEM46	0.025312625	5.49E-11	1.67E-08	0.99999984
IFI27	0.025057033	8.55E-11	2.47E-08	0.99999977
ANKDD1A	0.024714807	1.53E-10	4.16E-08	0.99999963
GPR44	0.024651292	1.71E-10	4.57E-08	0.99999959
ZNF462	0.024599166	1.87E-10	4.95E-08	0.99999956
SMC6L1	0.024376158	2.71E-10	6.89E-08	0.99999945
SDK2	0.024105571	4.26E-10	1.03E-07	0.99999914
COL5A3	0.023975305	5.29E-10	1.24E-07	0.9999989
IGFBP3	0.023716549	8.08E-10	1.81E-07	0.99999852
IL17RB	0.023654901	8.94E-10	1.97E-07	0.99999826
CCRN4L	-0.023485967	1.18E-09	2.51E-07	0.999998
FLJ32130	-0.023267078	1.67E-09	3.42E-07	0.99999717
NDUFB1	-0.023225801	1.79E-09	3.62E-07	0.99999696
DHRS10	-0.022380114	6.75E-09	1.16E-06	0.99999085
C10orf4	-0.022287092	7.79E-09	1.32E-06	0.99998989
PNMA5	0.022176923	9.22E-09	1.53E-06	0.9999884
3-Mar	-0.021871473	1.47E-08	2.29E-06	0.99998332
C1orf61	0.02182735	1.57E-08	2.43E-06	0.99998332
PDE4A	0.021822484	1.58E-08	2.44E-06	0.99998205
PKN3	0.021784305	1.67E-08	2.56E-06	0.99998205

Table 2: List of 306 genes with direct edges with IL12RB2 in CAMP

TRIM59	0.021401872	2.96E-08	4.20E-06	0.9999684
IGSF2	0.021199534	3.99E-08	5.43E-06	0.99995834
RAP1A	0.021187483	4.06E-08	5.52E-06	0.99995834
IL9R	-0.021141088	4.35E-08	5.85E-06	0.99995433
MYOM2	-0.02104937	4.97E-08	6.57E-06	0.99995433
AKAP11	0.021001998	5.33E-08	6.97E-06	0.99994871
C9orf79	-0.020850509	6.64E-08	8.42E-06	0.99994182
WDR77	-0.020717877	8.03E-08	9.91E-06	0.9999281
TCEAL2	0.020497675	1.10E-07	1.30E-05	0.9999126
PGEA1	0.020443386	1.19E-07	1.38E-05	0.99990911
POMZP3	0.020418606	1.23E-07	1.43E-05	0.99990242
SIPA1L2	0.020372121	1.31E-07	1.51E-05	0.99989447
LILRA1	-0.020337147	1.38E-07	1.57E-05	0.99989333
NPB	-0.020314592	1.43E-07	1.62E-05	0.99987727
PTRF	-0.020242689	1.58E-07	1.76E-05	0.99986603
ZNF532	0.020146302	1.81E-07	1.98E-05	0.99985992
CT45-2	-0.020028573	2.13E-07	2.28E-05	0.99984748
MATN2	0.020011935	2.18E-07	2.33E-05	0.99983751
PLEKHK1	-0.01981951	2.84E-07	2.92E-05	0.99981576
TCF8	-0.019749556	3.13E-07	3.16E-05	0.99979246
CASP3	-0.019747829	3.14E-07	3.17E-05	0.99979246
PDE4DIP	0.019659933	3.54E-07	3.51E-05	0.99977475
ENTPD1	0.019624847	3.71E-07	3.65E-05	0.99976076
FLJ31659	-0.01954025	4.16E-07	4.03E-05	0.99973347
PDE1B	0.019504102	4.37E-07	4.20E-05	0.99973347
UBE2I	0.01949964	4.40E-07	4.22E-05	0.99972099
ACTR3B	-0.019469762	4.58E-07	4.37E-05	0.99971059
CCR3	0.019451644	4.70E-07	4.46E-05	0.99971059
AXL	0.019342969	5.44E-07	5.05E-05	0.99967275
JAK3	-0.019339315	5.46E-07	5.07E-05	0.99967275
TREX2	0.0192845	5.88E-07	5.39E-05	0.99964918
JUNB	-0.019231909	6.31E-07	5.72E-05	0.99964918
KIAA1967	0.019224767	6.37E-07	5.77E-05	0.99963183
SLC22A4	-0.01920853	6.51E-07	5.88E-05	0.99963183
ANKRD15	0.019182964	6.74E-07	6.05E-05	0.99960683
LOC283755	0.019164879	6.90E-07	6.17E-05	0.99960683
SF1	0.019130743	7.22E-07	6.41E-05	0.99960683
PTGDS	-0.019121942	7.31E-07	6.48E-05	0.99960683
PAQR6	0.019103776	7.49E-07	6.61E-05	0.9995935
ATHL1	-0.019099154	7.54E-07	6.64E-05	0.99955769
LOC401233	-0.019040825	8.14E-07	7.10E-05	0.99953204
FLJ31438	0.018920467	9.55E-07	8.12E-05	0.99950131
TMEM107	-0.018835245	1.07E-06	8.91E-05	0.99947021
ZP3	0.018748794	1.20E-06	9.81E-05	0.99936534
DPPA4	0.01871909	1.24E-06	0.000101338	0.99936534
ROBO3	-0.018681785	1.31E-06	0.000105559	0.99934936
MITF	-0.018665463	1.33E-06	0.000107453	0.99934936
C10orf39	-0.018664232	1.34E-06	0.000107597	0.99934936
FXYD5	-0.018613495	1.43E-06	0.000113654	0.99934936

Table 2: List of 306 genes with direct edges with IL12RB2 in CAMP

TAP2	0.018584095	1.48E-06	0.000117321	0.99928273
LGALS12	-0.018550638	1.55E-06	0.000121648	0.99927301
TTC10	-0.018444797	1.77E-06	0.000136244	0.99919561
CREB3L3	-0.018370069	1.95E-06	0.000147571	0.99915226
SULT1A2	0.018346599	2.01E-06	0.000151279	0.99909542
C18orf56	0.018327815	2.06E-06	0.000154335	0.99909542
ATF7IP	0.018324641	2.07E-06	0.000154855	0.99909542
C6orf191	-0.018275743	2.20E-06	0.000163121	0.99903825
MGP	0.018088209	2.80E-06	0.000198943	0.9987881
NT5DC2	0.018002584	3.12E-06	0.000217617	0.99873433
RPS6KA2	-0.017974489	3.23E-06	0.000224064	0.99868028
FXYD2	0.017934441	3.40E-06	0.000233623	0.99861197
NPTX1	-0.017930342	3.41E-06	0.000234624	0.99861197
CCNT2	-0.017894316	3.57E-06	0.00024356	0.99861197
NRCAM	0.017831765	3.86E-06	0.000259832	0.99849577
RFPL2	-0.017786632	4.08E-06	0.000272174	0.99839812
MMP9	0.017751119	4.27E-06	0.00028232	0.99837735
MGC3101	-0.017718996	4.44E-06	0.000291746	0.99837735
SMG6	0.017686161	4.63E-06	0.000301703	0.9982369
HLA-DPB1	0.017597105	5.17E-06	0.000330592	0.9980553
COL6A1	0.017563429	5.39E-06	0.000342133	0.9980553
OR10G8	-0.017539068	5.55E-06	0.000350673	0.99800595
TSGA10	-0.01746957	6.04E-06	0.000376157	0.99786436
MRE11A	-0.017451026	6.18E-06	0.000383244	0.99786436
PTCH	-0.017420938	6.41E-06	0.000395033	0.99775385
RAB40C	0.01737382	6.79E-06	0.000414194	0.99767046
ANKRD28	0.017345971	7.03E-06	0.000425872	0.99759402
THTPA	0.01726236	7.78E-06	0.000463127	0.9973953
SLC16A11	-0.017260425	7.80E-06	0.000464021	0.9973953
DNAI2	-0.01711599	9.28E-06	0.000535528	0.99706133
DNAJC1	0.017093977	9.53E-06	0.00054721	0.99704009
FGF3	0.017091103	9.56E-06	0.000548751	0.99704009
LSS	0.017088825	9.59E-06	0.000549974	0.99704009
MGC24975	0.017045521	1.01E-05	0.000573852	0.99685895
C6orf25	-0.017010267	1.05E-05	0.000593899	0.99685895
COL25A1	0.016965215	1.11E-05	0.000620549	0.99669769
KCNH6	-0.016937059	1.15E-05	0.000637765	0.99646156
CLEC10A	-0.016915259	1.18E-05	0.000651439	0.99646156
SH3MD2	-0.016895338	1.21E-05	0.000664132	0.99635277
GPR113	-0.016869439	1.25E-05	0.000681009	0.99633468
OGDH	0.0167753	1.39E-05	0.000746186	0.99588135
SH3KBP1	-0.016736937	1.46E-05	0.000774411	0.9956887
DBF4B	0.016573491	1.76E-05	0.000905473	0.9951256
ARFIP2	0.01653687	1.84E-05	0.000937465	0.99504024
LASS4	-0.016498271	1.93E-05	0.0009722	0.99495799
ENG	0.016436429	2.07E-05	0.001030181	0.99459755
MXD3	-0.016378101	2.21E-05	0.001088894	0.99392114
KIAA0265	0.016360349	2.26E-05	0.001107325	0.99392114
FANK1	0.016335971	2.32E-05	0.001132999	0.99392114

Table 2: List of 306 genes with direct edges with IL12RB2 in CAMP

RGS1	0.016332476	2.33E-05	0.001136714	0.99392114
ZNF658B	0.016306987	2.40E-05	0.001164073	0.99366903
CA1	0.016292923	2.44E-05	0.001179523	0.99362803
B3Gn-T6	-0.016226567	2.64E-05	0.001255189	0.99320729
TSPAN33	-0.016168871	2.82E-05	0.001324365	0.99292564
LOC255374	-0.016166594	2.82E-05	0.001327163	0.99292564
APPBP1	-0.016164473	2.83E-05	0.001329775	0.99292564
C22orf8	-0.016155856	2.86E-05	0.001340419	0.99292564
ACSBG2	-0.016134242	2.93E-05	0.001367385	0.99292564
C12orf60	-0.016117188	2.99E-05	0.001389088	0.99254072
OTEX	-0.016065184	3.17E-05	0.00145743	0.99217326
AFMID	0.01600249	3.40E-05	0.001543951	0.99199943
DHFR	0.016000327	3.41E-05	0.001547	0.99199943
BCR	0.015957296	3.58E-05	0.001608707	0.99189544
PLA2G7	0.015951006	3.60E-05	0.00161787	0.99189544
KRTCAP3	0.015945812	3.63E-05	0.001625462	0.99189544
HOXA7	-0.01593291	3.68E-05	0.00164443	0.99189544
INSIG1	-0.015912715	3.76E-05	0.001674685	0.9911138
PCTP	0.015847157	4.05E-05	0.001777175	0.99087014
SLAMF7	0.015786085	4.34E-05	0.001877485	0.99032315
ITPKC	0.015762757	4.45E-05	0.001917187	0.99032315
P2RX5	0.015758148	4.47E-05	0.001925099	0.99032315
C14orf161	0.01568675	4.84E-05	0.002052351	0.98899484
NT5E	-0.015681773	4.87E-05	0.002061591	0.98899484
DEFA4	-0.015667933	4.95E-05	0.00208744	0.98899484
RFC3	-0.015572988	5.49E-05	0.002271866	0.98852772
IL27	0.015558837	5.58E-05	0.002300426	0.98826293
C18orf24	0.015522291	5.81E-05	0.00237603	0.98807711
MGC32020	0.015425016	6.46E-05	0.002589927	0.98679433
HLF	-0.015407555	6.59E-05	0.002629886	0.98679433
GCC2	0.015398917	6.65E-05	0.002649809	0.98679433
TAF1A	0.015376565	6.81E-05	0.00270188	0.98674977
RBPMS2	0.015372055	6.85E-05	0.002712471	0.98674977
RBMS2	0.015370903	6.86E-05	0.002715179	0.98674977
NRG1	0.015330836	7.16E-05	0.002810561	0.98674977
MEIS1	-0.015249413	7.82E-05	0.003013417	0.98494957
MELK	0.015236007	7.94E-05	0.003048726	0.98438932
TNFAIP6	0.015233352	7.96E-05	0.003055777	0.98438932
LOC441168	0.015182866	8.41E-05	0.003192157	0.98425405
CDKL3	-0.015153732	8.67E-05	0.003273067	0.98362848
TRIM47	-0.015145586	8.75E-05	0.003296077	0.98362848
TMPRSS3	-0.015080861	9.38E-05	0.003482788	0.98362848
GRIPAP1	0.015030759	9.90E-05	0.003633491	0.98241755
PAPPA2	0.014969247	0.00010568	0.003826532	0.98216388
DKFZp686O24	0.014967958	0.00010582	0.003830655	0.98216388
MYO1C	0.014913078	0.00011217	0.004011391	0.98111739
JAKMIP2	-0.014912089	0.00011229	0.004014708	0.98111739
LOC201175	0.014897691	0.00011402	0.004063203	0.98106757
FKBPL	0.014839087	0.00012131	0.004265929	0.97960458

Table 2: List of 306 genes with direct edges with IL12RB2 in CAMP

MDGA1	-0.014831696	0.00012226	0.004292312	0.97960458
FAM112A	-0.014826975	0.00012287	0.004309224	0.97960458
SHD	-0.014821626	0.00012357	0.004328435	0.97960458
THAP8	-0.014819525	0.00012384	0.004335995	0.97960458
BFSP2	0.014815747	0.00012434	0.004349615	0.97960458
LOC91431	-0.014764018	0.0001313	0.004539003	0.97930998
TTK	0.014749532	0.00013331	0.004593212	0.97914006
LOC196463	-0.014723312	0.00013703	0.004693141	0.97818009
FLCN	0.014701492	0.0001402	0.00477829	0.97665773
PPAP2C	-0.014661291	0.00014622	0.004940062	0.97664075
ZNF80	0.014652407	0.00014758	0.0049763	0.97664075
RAB14	-0.014616584	0.0001532	0.005124357	0.97587957
TNFRSF10D	-0.01455873	0.0001627	0.005371856	0.97484871
FAAH	0.014533286	0.00016705	0.005484199	0.97484871
LOC200810	-0.014524775	0.00016853	0.005522364	0.97346503
TNFSF13B	0.014517668	0.00016978	0.005554622	0.97346503
KIF27	-0.014473838	0.00017765	0.00575721	0.97241494
RPRM	-0.014453133	0.00018149	0.005855165	0.97241494
AMDHD1	-0.014425348	0.00018676	0.005988452	0.97228056
FLJ23834	0.014398425	0.000192	0.006120575	0.970929
TNFRSF17	-0.014329949	0.00020597	0.006466859	0.970929
LMNA	0.014267828	0.00021947	0.006795932	0.96891429
PPFIBP2	-0.014265279	0.00022004	0.006809743	0.96891429
KIF1B	0.014249079	0.0002237	0.00689793	0.96891429
SIGLEC5	-0.014227969	0.00022856	0.007014006	0.96877352
DACT1	-0.014110644	0.00025742	0.007692275	0.96549624
PHF6	0.014068558	0.00026857	0.00795048	0.96427136
CCR10	-0.014032045	0.00027862	0.008180984	0.96177263
DUSP10	-0.014000425	0.0002876	0.008387173	0.96117821
CAMK2N1	-0.013994626	0.00028928	0.00842546	0.96117821
IGFL2	0.013987154	0.00029146	0.008474957	0.96117821
KISS1R	-0.013964976	0.000298	0.00862296	0.96117821
FMNL3	-0.0139572	0.00030032	0.008675238	0.96117821
CPA5	0.013951022	0.00030219	0.008716914	0.96117821
TBKBP1	0.013935	0.00030706	0.00882558	0.96117821
MMP11	-0.013879967	0.00032437	0.009206038	0.96083964
SDSL	0.01384338	0.00033639	0.009467502	0.95698015
KRTHA7	-0.013840113	0.00033748	0.009491466	0.95698015
INDOL1	-0.013839166	0.0003378	0.009498417	0.95698015
PDF	0.013836069	0.00033884	0.009521185	0.95698015
DMXL2	-0.013788324	0.00035525	0.009876513	0.9569218
TXNDC5	-0.013783739	0.00035686	0.00991107	0.9569218
ME3	0.013767298	0.00036271	0.010035614	0.9569218
TMEM110	-0.01375205	0.00036821	0.010152076	0.95652661
KIAA1446	0.013728162	0.00037699	0.010336521	0.95652661
DUSP4	0.013724259	0.00037844	0.010366851	0.95652661
VSIG4	-0.013718458	0.00038061	0.01041217	0.95331437
C20orf22	-0.013702391	0.00038667	0.010541053	0.95281958
UBE2A	0.013689019	0.00039179	0.010649075	0.95281958

Table 2: List of 306 genes with direct edges with IL12RB2 in CAMP

CHX10	-0.013641047	0.00041068	0.011043513	0.95218596
TRIM51	0.013606222	0.00042492	0.011336706	0.94981569
MAOB	0.013559224	0.00044486	0.011745107	0.94939015
PTPRE	-0.013550005	0.00044888	0.011826322	0.94939015
UPB1	-0.013469857	0.00048523	0.012563735	0.94456399
ZNF415	0.013436142	0.00050132	0.012884547	0.94367339
SASS6	-0.013431942	0.00050336	0.012924934	0.94367339
GSTM1	-0.013424576	0.00050696	0.012995959	0.94367339
PIWIL4	0.013423255	0.0005076	0.013008729	0.94367339
MICALCL	-0.01341771	0.00051033	0.013062378	0.94367339
C8orf58	-0.013388288	0.00052502	0.013349336	0.94367339
FAM14A	-0.013380836	0.00052881	0.013422611	0.94367339
PDGFC	-0.013377047	0.00053074	0.013459968	0.94367339
RFP2	-0.013361092	0.00053895	0.013619074	0.94126085
CTRC	-0.013359861	0.00053959	0.013631463	0.94126085
ASB6	0.013353645	0.00054283	0.013694159	0.94064305
FLJ45645	0.013352051	0.00054366	0.013710321	0.94044209
ANKK1	0.013334876	0.00055271	0.013885293	0.94044209
KDELR1	-0.013257241	0.00059538	0.014695146	0.9390541
ZSCAN2	-0.013245401	0.00060215	0.014821639	0.9390541
BRRN1	0.013239858	0.00060535	0.014881077	0.9390541
DSCAM	0.013236441	0.00060733	0.014917785	0.9390541
FAM20C	0.013226594	0.00061306	0.015023859	0.9390541
FCRL2	-0.013220084	0.00061688	0.01509423	0.9390541
CDA	-0.013200493	0.0006285	0.015307151	0.9390541
C21orf58	0.01319007	0.00063476	0.015421132	0.9390541
ARRDC2	0.013173811	0.00064465	0.015600872	0.93798965
HP	-0.013172042	0.00064573	0.015620503	0.93798965
PLEC1	0.013151972	0.00065816	0.015847682	0.93169159
C9orf9	-0.013146564	0.00066154	0.015910204	0.93169159
LOC400707	0.013144548	0.00066281	0.01593356	0.93169159
NALP7	-0.013142408	0.00066416	0.01595837	0.93169159
CD209	0.013132191	0.00067062	0.016077357	0.93077517
MSC	0.013099755	0.00069153	0.016459467	0.93077517
GNAT2	0.01306788	0.00071267	0.016840322	0.92850682
FLJ20130	0.013032583	0.00073677	0.01727313	0.92774568
NIPSNAP3A	-0.013025822	0.00074148	0.017356898	0.92774568
C3orf17	0.012947761	0.00079784	0.018342909	0.92604417
NDN	0.012906616	0.00082912	0.018886267	0.92168872
BCL11A	-0.012903154	0.0008318	0.018932603	0.92168872
CD151	0.012890156	0.00084195	0.01910721	0.92168872
TSPAN32	-0.012873532	0.00085509	0.019331869	0.92168872
DDX3Y	-0.012830053	0.00089037	0.019926993	0.92137441
KIAA0125	0.012813181	0.00090442	0.020161083	0.91904647
CFH	0.012798511	0.00091681	0.02036853	0.91796305
CXCL10	0.012787713	0.00092602	0.020522568	0.91705485
GPR34	0.012740904	0.00096697	0.021199946	0.91571762
TNFRSF13B	0.012740248	0.00096756	0.02120955	0.91571762
ARHGEF4	0.012694931	0.00100882	0.02188358	0.91306965

Table 2: List of 306 genes with direct edges with IL12RB2 in CAMP

ANKRD17	-0.012688222	0.00101507	0.021984775	0.91306965
PRG4	-0.012659454	0.00104225	0.022424599	0.90736408
DACH1	0.012655221	0.00104631	0.022490691	0.90736408
SMCX	0.012650091	0.00105125	0.022570947	0.90736408
HDGFRP3	0.012643582	0.00105754	0.022672998	0.90736408
KARCA1	-0.012634741	0.00106614	0.022812046	0.90736408
ALOX5	-0.012593717	0.00110692	0.023463596	0.90736408
ALG10	-0.012559962	0.00114155	0.024008283	0.906688
ENTPD6	-0.01254282	0.00115952	0.024287812	0.906688
SYNC1	0.012536871	0.00116582	0.024385232	0.906688
TGIF	-0.012530921	0.00117215	0.024484505	0.90113368
PCP2	-0.012497512	0.00120828	0.025048018	0.90113368
SS18	0.012494436	0.00121166	0.025100263	0.90113368
