# Supplementary material for: A graphical model approach for inferring large-scale networks integrating gene expression and genetic polymorphism
Source: BMC Syst Biol. 2009 May 27;3:55. doi: 10.1186/1752-0509-3-55 (PMC2694152; doi:10.1186/1752-0509-3-55)
Supplement: Additional file 3 — List of 225 significant gene-SNP associations in CAMP connected to IL12RB2. This table includes 225 significantly associated gene-SNP pairs where the gene is connected to IL12RB2 in the gene-gene network in CAMP dataset. [file 1752-0509-3-55-S3.pdf]

Table 3: List of 225 significant gene-SNP associations in CAMP connected to IL12RB2

Gene	Marker	FDR adjusted p-value
QRSL1	rs2054365	1.96E-09
QRSL1	rs1419055	6.30E-10
QRSL1	rs7750365	0.00E+00
QRSL1	rs3804345	0.00E+00
QRSL1	rs3127657	0.00E+00
QRSL1	rs3101493	0.00E+00
QRSL1	rs3121447	1.69E-10
QRSL1	rs3121445	0.00E+00
QRSL1	rs3104037	3.22E-04
DPYSL4	rs870552	2.98E-06
DPYSL4	rs4880241	8.68E-06
DPYSL4	rs7915260	1.69E-05
DPYSL4	rs4880341	8.68E-06
DPYSL4	rs1873504	2.10E-05
DPYSL4	rs2101613	5.90E-06
DPYSL4	rs923662	7.01E-05
DPYSL4	rs2818405	4.96E-02
DPYSL4	rs2035804	3.48E-04
PRSS16	rs6938200	1.15E-02
HOXB4	rs11079828	2.22E-05
HOXB4	rs9905940	1.56E-05
HOXB4	rs6504340	1.56E-05
HOXB4	rs1042815	6.15E-05
HOXB4	rs2229302	5.03E-03
HOXB4	rs890432	1.07E-03
HOXB4	rs2555111	2.26E-02
HOXB4	rs2288278	1.56E-02
HOXB4	rs9299	3.55E-03
RNF175	rs10517577	1.20E-03
RNF175	rs2606324	2.03E-04
RNF175	rs2251997	2.03E-04
RNF175	rs2251900	2.03E-04
RNF175	rs11099898	4.17E-06
RNF175	rs1598632	3.53E-06
RNF175	rs7653960	1.84E-04
SLC25A29	rs8021068	8.56E-06
SLC25A29	rs1059264	0.00E+00
SLC25A29	rs1059263	2.53E-03
SLC25A29	rs8007427	8.12E-12
WWC1	rs4976597	1.06E-04
WWC1	rs4976598	3.73E-05
TMCC1	rs1136553	1.00E-09
TMCC1	rs2811425	1.27E-04
TMCC1	rs4688772	1.54E-08
ANKDD1A	rs1628955	0.00E+00
ANKDD1A	rs936867	9.47E-10
ANKDD1A	rs1471834	1.21E-04
ANKDD1A	rs2056497	1.42E-02

Table 3: List of 225 significant gene-SNP associations in CAMP connected to IL12RB2

ANKDD1A	rs4287512	7.01E-05
SMC6L1	rs4578856	3.64E-08
SMC6L1	rs7556886	3.39E-07
COL5A3	rs2303099	1.26E-03
IGFBP3	rs2471552	2.08E-03
IGFBP3	rs2965073	3.62E-03
IGFBP3	rs1534151	6.07E-03
IGFBP3	rs1534150	4.76E-03
NDUFB1	rs3814833	0.00E+00
NDUFB1	rs12586783	2.31E-05
NDUFB1	rs4548800	7.09E-03
NDUFB1	rs2025072	6.71E-03
DHRS10	rs8110220	5.59E-07
DHRS10	rs16982311	1.19E-03
RAP1A	rs10489469	9.38E-03
RAP1A	rs3767595	8.89E-03
MYOM2	rs1866717	4.36E-04
MYOM2	rs6983960	1.67E-04
MYOM2	rs7002629	4.78E-02
MYOM2	rs2280899	5.09E-03
MYOM2	rs2280904	7.69E-04
WDR77	rs1264874	2.66E-02
WDR77	rs2485319	4.76E-02
WDR77	rs12040764	3.28E-02
POMZP3	rs17718122	0.00E+00
POMZP3	rs1754183	6.50E-13
CASP3	rs1405940	4.64E-02
CASP3	rs2696051	7.65E-03
ATHL1	rs2242565	1.22E-06
LOC401233	rs927340	1.14E-06
LOC401233	rs911536	2.10E-06
LOC401233	rs1885300	1.38E-06
LOC401233	rs7762140	2.52E-11
LOC401233	rs1040521	0.00E+00
LOC401233	rs7774283	0.00E+00
LOC401233	rs7761969	0.00E+00
LOC401233	rs7765538	1.74E-05
LOC401233	rs7775816	2.38E-03
LOC401233	rs12200314	0.00E+00
FLJ31438	rs7349405	1.27E-07
FLJ31438	rs4435493	2.71E-07
ZP3	rs2286427	2.66E-02
ZP3	rs2286428	1.05E-03
ROBO3	rs11219821	3.53E-08
ROBO3	rs4606490	7.24E-04
ROBO3	rs4586175	7.19E-03
ROBO3	rs4077566	8.42E-08
C6orf191	rs2326871	8.82E-03
C6orf191	rs9402178	2.10E-05

Table 3: List of 225 significant gene-SNP associations in CAMP connected to IL12RB2

C6orf191	rs7776426	2.61E-05
C6orf191	rs9492393	1.80E-08
C6orf191	rs9321191	2.98E-07
C6orf191	rs1480513	2.10E-05
C6orf191	rs9402186	2.92E-05
C6orf191	rs9398926	8.56E-06
C6orf191	rs9321194	9.30E-08
C6orf191	rs6939565	2.55E-05
C6orf191	rs10499166	8.68E-06
C6orf191	rs1337778	2.53E-03
C6orf191	rs9375674	5.88E-04
MGP	rs918121	1.25E-02
MGP	rs10772817	1.25E-02
MGP	rs10846082	1.25E-02
MGP	rs10047541	2.55E-02
MGP	rs2193360	1.25E-02
MGP	rs2445370	8.84E-03
NT5DC2	rs1010554	3.97E-02
NPTX1	rs8071962	4.29E-02
MGC3101	rs3803684	1.51E-03
MGC3101	rs7498985	1.01E-02
SLC16A11	rs189772	3.43E-02
APPBP1	rs363175	3.12E-04
APPBP1	rs422945	3.12E-04
C22orf8	rs104664	0.00E+00
C22orf8	rs742013	5.88E-03
C22orf8	rs6007594	5.88E-03
C22orf8	rs136564	0.00E+00
BCR	rs5751611	4.80E-02
BCR	rs2156921	1.25E-02
BCR	rs131678	1.36E-02
BCR	rs2239666	3.50E-02
KRTCAP3	rs704791	7.22E-08
KRTCAP3	rs1260342	7.22E-08
KRTCAP3	rs4803	7.22E-08
KRTCAP3	rs780104	1.85E-07
KRTCAP3	rs780106	1.85E-07
KRTCAP3	rs1647276	7.22E-08
KRTCAP3	rs1647266	9.70E-08
KRTCAP3	rs1260345	4.06E-06
KRTCAP3	rs2272417	6.01E-06
INSIG1	rs13223383	4.31E-03
MDGA1	rs12191311	3.73E-05
MDGA1	rs10947686	1.85E-06
MDGA1	rs4714089	3.27E-09
MDGA1	rs7745284	6.16E-03
MDGA1	rs9462343	2.14E-04
MDGA1	rs3846876	1.96E-09
MDGA1	rs4711509	8.68E-10

Table 3: List of 225 significant gene-SNP associations in CAMP connected to IL12RB2

MDGA1	rs10947690	6.48E-03
MDGA1	rs6925749	3.89E-09
MDGA1	rs6903441	4.72E-04
MDGA1	rs4714092	2.22E-06
MDGA1	rs10947692	3.83E-03
MDGA1	rs989713	8.43E-06
MDGA1	rs11752386	1.29E-02
MDGA1	rs6916856	1.21E-07
MDGA1	rs6458011	5.08E-10
MDGA1	rs10947693	2.29E-03
MDGA1	rs6938061	0.00E+00
ZNF80	rs3732781	4.05E-02
ZNF80	rs1354350	1.33E-03
RAB14	rs3789311	6.06E-07
RPRM	rs4294952	3.03E-02
AMDHD1	rs10161146	7.99E-08
AMDHD1	rs2060548	2.19E-03
AMDHD1	rs10735337	9.45E-03
AMDHD1	rs4762254	2.37E-03
AMDHD1	rs726869	2.06E-03
AMDHD1	rs10859990	6.06E-03
AMDHD1	rs4762651	1.34E-04
AMDHD1	rs1025607	4.39E-04
PPFIBP2	rs4322372	2.79E-10
PPFIBP2	rs4757999	7.81E-10
PPFIBP2	rs7925442	2.10E-05
PPFIBP2	rs4758001	6.30E-11
PPFIBP2	rs7481343	1.37E-05
PPFIBP2	rs4076808	1.19E-02
KISS1R	rs2965292	1.09E-12
CPA5	rs2171492	5.45E-03
CPA5	rs3800775	5.18E-03
CPA5	rs7793804	6.37E-03
CPA5	rs7798773	6.37E-03
CPA5	rs10234851	6.16E-03
CPA5	rs888397	9.89E-11
CPA5	rs1544705	0.00E+00
CPA5	rs17164851	1.15E-12
CPA5	rs11761888	0.00E+00
CPA5	rs748812	6.65E-08
CPA5	rs11772922	0.00E+00
CPA5	rs17164872	6.40E-06
CPA5	rs968404	1.26E-09
CPA5	rs12706927	3.89E-09
CPA5	rs17330508	1.84E-06
TBKBP1	rs11870935	2.64E-02
TBKBP1	rs6503796	6.26E-04
TBKBP1	rs4794053	6.53E-04
TBKBP1	rs9913503	8.21E-04

Table 3: List of 225 significant gene-SNP associations in CAMP connected to IL12RB2

MMP11	rs2070454	4.13E-04
ME3	rs1540082	3.25E-02
ME3	rs10400291	4.20E-02
TMEM110	rs1986656	7.86E-03
KIAA1446	rs7142209	2.60E-10
KIAA1446	rs4074037	2.77E-12
KIAA1446	rs10148448	3.92E-04
KIAA1446	rs7493101	1.79E-02
C20orf22	rs2274890	2.20E-09
C20orf22	rs6083828	0.00E+00
UPB1	rs738813	1.40E-11
UPB1	rs5760452	5.47E-12
UPB1	rs3788372	2.42E-04
UPB1	rs738814	4.44E-03
UPB1	rs6004171	7.44E-04
ZNF415	rs10423215	2.23E-04
GSTM1	rs11101992	3.28E-02
LOC400707	rs8109759	5.07E-05
LOC400707	rs2311223	9.66E-06
LOC400707	rs10416550	1.15E-12
LOC400707	rs8107491	5.71E-11
LOC400707	rs877589	5.10E-13
LOC400707	rs2288913	1.88E-05
LOC400707	rs7254929	0.00E+00
LOC400707	rs8112461	7.22E-08
LOC400707	rs6509283	2.01E-05
LOC400707	rs10408465	4.42E-05
LOC400707	rs3745795	6.06E-03
CD151	rs3059	2.03E-02
TSPAN32	rs16928081	1.31E-02
