# Supplementary material for: A graphical model approach for inferring large-scale networks integrating gene expression and genetic polymorphism
Source: BMC Syst Biol. 2009 May 27;3:55. doi: 10.1186/1752-0509-3-55 (PMC2694152; doi:10.1186/1752-0509-3-55)
Supplement: Additional file 4 — List of 353 significant gene-SNP associations in CAMP connected to IL1B. This table includes 353 significantly associated gene-SNP pairs where the gene is connected to IL1B in the gene-gene network in CAMP dataset. [file 1752-0509-3-55-S4.pdf]

Table 4: List of 353 significant gene-SNP associations in CAMP connected to IL1B

Gene	Marker	FDR adjusted p-value
C20orf22	rs2268879	1.30E-07
C20orf22	rs6083776	0
C20orf22	rs2076559	4.65E-11
C20orf22	rs1007707	0
C20orf22	rs1980497	0.03606998
C20orf22	rs11699316	6.11E-05
C20orf22	rs3746337	0
C20orf22	rs11697384	1.43E-08
C20orf22	rs2274890	8.27E-10
C20orf22	rs6083828	0
C20orf22	rs2482911	0
C20orf22	rs6076347	0
C20orf22	rs2500406	0
C20orf22	rs4423675	0
C20orf22	rs3764694	0.00512132
C20orf22	rs8115804	1.34E-08
C20orf22	rs6138575	0
C20orf22	rs417110	0
C20orf22	rs2281212	3.33E-05
C20orf22	rs404775	0
PPFIBP2	rs4408288	0.00385343
PPFIBP2	rs10769793	0.00064841
PPFIBP2	rs3884596	0.00168512
PPFIBP2	rs4078258	0.00105432
PPFIBP2	rs10839797	0.00240555
PPFIBP2	rs4322372	2.48E-11
PPFIBP2	rs4757999	7.79E-11
PPFIBP2	rs7925442	4.58E-06
PPFIBP2	rs4758001	9.28E-13
PPFIBP2	rs6578869	0.02040056
PPFIBP2	rs7481343	2.63E-06
PPFIBP2	rs4076808	0.00474473
PPFIBP2	rs7942861	0.04169588
PILRB	rs3735241	0.04187667
PILRB	rs6955367	0
PILRB	rs4074838	0
PILRB	rs6955362	0
PILRB	rs7806537	8.77E-05
PILRB	rs4463351	0
PILRB	rs11761624	9.86E-11
NR2F6	rs2288540	0.00836563
NR2F6	rs7258100	0.00906012
NR2F6	rs2288539	0.00286541
NR2F6	rs9676419	0.04839542
WBSCR27	rs1059779	0.01924197
WBSCR27	rs6460052	1.73E-05
WBSCR27	rs11764572	8.20E-06
WBSCR27	rs12536661	2.76E-11

Table 4: List of 353 significant gene-SNP associations in CAMP connected to IL1B

WBSCR27	rs6460055	0
WBSCR27	rs6979031	5.35E-06
WBSCR27	rs13232536	0
WBSCR27	rs9647713	7.18E-10
WBSCR27	rs4255023	0
WBSCR27	rs4304218	0
WBSCR27	rs4717112	0
WBSCR27	rs13244770	0
PLA2G4C	rs1653554	2.17E-05
PLA2G4C	rs251693	2.10E-06
PLA2G4C	rs2304136	0.00419766
PLA2G4C	rs3730913	6.45E-05
PLA2G4C	rs274860	0.00015314
PLA2G4C	rs274873	0.00030084
PLA2G4C	rs10405655	0.00619274
PLA2G4C	rs2007183	0.0002425
CHGB	rs13043139	0.02649981
CHGB	rs4813777	4.88E-06
CHGB	rs505528	1.11E-05
CHGB	rs1555141	0.00356692
CHGB	rs1151948	0
CHGB	rs415023	0
IGSF4	rs4938180	0.00010805
IGSF4	rs4936322	0.00369867
IGSF4	rs7101437	6.36E-05
IGSF4	rs6589485	0.00300955
IGSF4	rs11602686	0.00812705
BLK	rs12680762	0.02039191
BLK	rs2736340	3.02E-05
BLK	rs13277113	6.45E-05
BLK	rs12677843	0.0207876
BLK	rs2248932	0.04649119
GSTM1	rs2269340	0.0002276
GSTM1	rs7544426	0.0263939
CTSK	rs11204684	0.00037676
CTSK	rs4970926	0.00030084
CTSK	rs6694531	0.00028303
CTSK	rs7412746	0.00018161
CTSK	rs3754211	0.00015683
CTSK	rs2305814	0.00202067
HLA-DRB5	rs9368713	0.00143568
HLA-DRB5	rs9405090	0.00143568
HLA-DRB5	rs1033500	0.0011497
HLA-DRB5	rs910049	1.21E-11
HLA-DRB5	rs1265759	0.00020593
HLA-DRB5	rs1265758	5.31E-05
HLA-DRB5	rs2395150	4.89E-05
HLA-DRB5	rs9268368	0.00143568
HLA-DRB5	rs3129934	0

Table 4: List of 353 significant gene-SNP associations in CAMP connected to IL1B

HLA-DRB5	rs9268384	0.00152407
HLA-DRB5	rs3129941	3.11E-12
HLA-DRB5	rs2395157	0.01106582
HLA-DRB5	rs4424066	0.00121967
HLA-DRB5	rs3117098	6.11E-09
HLA-DRB5	rs3817973	0.00121967
HLA-DRB5	rs2076530	0.0006809
HLA-DRB5	rs3817963	0.01596644
HLA-DRB5	rs3806156	0.0029768
HLA-DRB5	rs3763309	0.04966684
HLA-DRB5	rs6932542	5.28E-06
HLA-DRB5	rs9268528	9.44E-05
HLA-DRB5	rs9268542	9.44E-05
HLA-DRB5	rs3129860	0
HLA-DRB5	rs3135338	8.65E-05
HLA-DRB5	rs9268615	0.04759151
HLA-DRB5	rs2395173	8.65E-05
HLA-DRB5	rs3129871	0.00013355
HLA-DRB5	rs3129882	0.0083133
HLA-DRB5	rs2239804	0.00623769
HLA-DRB5	rs7192	3.43E-06
HLA-DRB5	rs2395182	3.82E-10
HLA-DRB5	rs9268832	6.36E-13
HLA-DRB5	rs6903608	6.71E-12
HLA-DRB5	rs2395185	0.00342049
HLA-DRB5	rs477515	0.00132516
HLA-DRB5	rs2516049	0.00132516
HLA-DRB5	rs660895	0.02082696
HLA-DRB5	rs532098	2.68E-08
HLA-DRB5	rs9271366	0
HLA-DRB5	rs9271568	0.00132516
HLA-DRB5	rs1063355	1.09E-09
HLA-DRB5	rs9275141	5.57E-05
HLA-DRB5	rs2856683	0.02039191
HLA-DRB5	rs7774434	0.00018792
HLA-DRB5	rs2157051	6.36E-13
HLA-DRB5	rs9275224	4.46E-05
HLA-DRB5	rs5000634	0.0019121
HLA-DRB5	rs6457617	4.38E-05
HLA-DRB5	rs2647012	2.28E-08
HLA-DRB5	rs9275312	0.03226207
HLA-DRB5	rs2856725	4.17E-08
HLA-DRB5	rs9275328	0.04035937
HLA-DRB5	rs9275371	0.00356692
HLA-DRB5	rs9275374	0.00356692
HLA-DRB5	rs9275388	0.00678827
HLA-DRB5	rs9275390	0.00229894
HLA-DRB5	rs9275393	0.00356692
HLA-DRB5	rs9275406	0.00356692

Table 4: List of 353 significant gene-SNP associations in CAMP connected to IL1B

HLA-DRB5	rs9275407	0.00356692
HLA-DRB5	rs9275408	0.00216573
HLA-DRB5	rs9275418	0.00356692
HLA-DRB5	rs2856717	4.17E-08
HLA-DRB5	rs2858305	4.17E-08
HLA-DRB5	rs9275424	0.00356692
HLA-DRB5	rs9275425	0.00356692
HLA-DRB5	rs9275427	0.00356692
HLA-DRB5	rs9275428	0.00356692
HLA-DRB5	rs9275439	0.00356692
HLA-DRB5	rs9275555	0.01590236
HLA-DRB5	rs9275563	0.00132516
HLA-DRB5	rs9275572	5.08E-06
HLA-DRB5	rs9275582	0.02172597
HLA-DRB5	rs2858332	5.31E-05
HLA-DRB5	rs9275595	0.02172597
HLA-DRB5	rs9275596	4.81E-09
HLA-DRB5	rs3104402	1.17E-11
HLA-DRB5	rs3104405	0.00116982
GSTM3	rs3754446	0.02447737
GSTM3	rs11807	4.11E-06
GSTM3	rs11101992	9.89E-08
GSTM3	rs4970774	0
GSTM3	rs1927328	2.75E-05
GSTM3	rs7483	7.38E-05
GSTM3	rs10735234	0
GSTM3	rs2274536	2.53E-06
GSTM3	rs1887546	1.96E-06
GSTM3	rs6684608	0
GSTM3	rs11591171	0.03716957
GSTM3	rs2094469	0.00273581
GSTM3	rs3818562	0.0010713
STX19	rs8178610	0.01916042
STX19	rs8178607	0
STX19	rs8178591	0
STX19	rs13062355	2.52E-06
STX19	rs7644769	0.01618042
STX19	rs6805571	0.00080221
STX19	rs4857242	0.02172597
KIAA1875	rs7831595	0.04869835
KIAA1875	rs6558406	0.04035937
KIAA1875	rs7819099	0.01596644
KIAA1875	rs6984820	0.03822858
KIAA1875	rs6993938	0.04869835
KIAA1875	rs7010330	0.03226207
KIAA1875	rs13264654	6.68E-12
KIAA1875	rs9650466	0.02493005
KIAA1875	rs12542298	0.0147229
KIR3DL2	rs11665986	3.65E-05

Table 4: List of 353 significant gene-SNP associations in CAMP connected to IL1B

KIR3DL2	rs269919	0.03471365
KIR3DL2	rs634742	0.00683845
KIR3DL2	rs269939	0.01891221
KIR3DL2	rs269940	0.01754774
KIR3DL2	rs269955	0.00161582
FLJ25791	rs1321206	0.03606998
FLJ25791	rs9384719	0.03894354
FLJ25791	rs9386829	0.03606998
NA	rs703817	0.00528549
NA	rs324015	0.01082109
NA	rs841718	0.01453489
FKSG14	rs2640717	0.00027012
FKSG14	rs6878202	0.00931061
FKSG14	rs7708972	0.00285541
FKSG14	rs786703	4.18E-07
FKSG14	rs1493451	0.00053891
FKSG14	rs33396	0
FKSG14	rs402552	0
FKSG14	rs380327	0
FKSG14	rs245566	0.00064841
FKSG14	rs245560	9.43E-09
FKSG14	rs6897886	0
FKSG14	rs6870248	0.00064841
FKSG14	rs149514	0
FKSG14	rs460262	0
FKSG14	rs36133	0
FKSG14	rs154861	0
FKSG14	rs16894099	0.00041902
FKSG14	rs468754	0
FKSG14	rs10057241	0.00432607
FKSG14	rs37349	0.00212215
FKSG14	rs37346	0
FKSG14	rs754784	0.00048275
FKSG14	rs28342	0.00533006
FKSG14	rs37329	0.00705694
FKSG14	rs37327	2.11E-07
FKSG14	rs112314	0
FKSG14	rs972501	0
LOC441046	rs11730153	0.00128432
LOC441046	rs1510881	9.90E-12
LOC441046	rs17017927	3.51E-10
LOC441046	rs10032932	0.01596644
LOC441046	rs2132935	0.01596644
CCL20	rs13389224	0.01948614
CCL20	rs11694155	0.00356692
ANKDD1A	rs3743046	2.63E-06
ANKDD1A	rs2414858	0
ANKDD1A	rs1522747	0
ANKDD1A	rs1352093	0

Table 4: List of 353 significant gene-SNP associations in CAMP connected to IL1B

ANKDD1A	rs832882	4.93E-06
ANKDD1A	rs832886	0.01328956
ANKDD1A	rs1628955	0
ANKDD1A	rs936867	5.44E-10
ANKDD1A	rs1471834	9.92E-05
ANKDD1A	rs2056497	0.01080647
ANKDD1A	rs4287512	8.77E-05
PALLD	rs1566498	0.0007847
C14orf132	rs1951300	0.03505784
C14orf132	rs8011771	0.02460504
C14orf132	rs8019430	0.00361167
C14orf132	rs11847484	0.00622821
C14orf132	rs10145060	7.27E-12
C14orf132	rs17093634	0.00011919
C14orf132	rs17093643	0.00076614
C14orf132	rs2208478	6.12E-11
C14orf132	rs1886059	1.00E-10
C14orf132	rs4274360	2.17E-10
C2orf26	rs892464	0.04169588
C2orf26	rs7590230	0.02172597
LOC401233	rs3823096	0.02736219
LOC401233	rs927340	2.28E-07
LOC401233	rs911536	1.48E-07
LOC401233	rs1885300	3.57E-07
LOC401233	rs7762140	8.78E-13
LOC401233	rs1040521	0
LOC401233	rs7774283	0
LOC401233	rs7761969	0
LOC401233	rs7765538	3.35E-06
LOC401233	rs7775816	0.00060913
LOC401233	rs12200314	0
LOC401233	rs6596945	0.00012164
C4BPB	rs6690037	0.00641149
HDGFRP3	rs2123157	0.03072132
HDGFRP3	rs716886	0.00121967
HDGFRP3	rs4531702	0.00043081
HDGFRP3	rs8042597	0.00073247
GSTT1	rs4822442	0.0113257
GSTT1	rs2236620	0.00376373
GSTT1	rs2070460	0.00177897
GSTT1	rs875643	0.00402683
GSTT1	rs738806	0.00528549
GSTT1	rs2012124	0.02641106
GSTT1	rs5760093	0.00121967
GSTT1	rs4820571	0
GSTT1	rs5760102	0
GSTT1	rs6003959	6.56E-10
GSTT1	rs4822458	0
GSTT1	rs1006771	3.94E-11

Table 4: List of 353 significant gene-SNP associations in CAMP connected to IL1B

GSTT1	rs5760147	0
GSTT1	rs140289	0.03397055
GSTT1	rs6004011	0.00011919
GSTT1	rs738809	3.31E-10
RPRM	rs11676810	0.03773
RPRM	rs4294952	0.02062101
RPRM	rs1595458	0.04818842
RPRM	rs4664144	0.04169588
SERPINE2	rs13396978	0.03927848
SERPINE2	rs3795877	0.00388754
SERPINE2	rs7590948	0.0011114
SERPINE2	rs2037755	0.00384802
SERPINE2	rs10194024	4.24E-07
SERPINE2	rs920251	0
SERPINE2	rs6719480	1.42E-08
SERPINE2	rs1438831	0
SERPINE2	rs282254	1.32E-07
SERPINE2	rs282258	0.00065974
SERPINE2	rs4674860	2.51E-05
SERPINE2	rs2196661	1.85E-11
SERPINE2	rs12694628	4.49E-09
RBMS2	rs2291738	0.04265038
RBMS2	rs3782235	0.02994297
RBMS2	rs774211	0.04994445
RBMS2	rs10876902	0.03659402
GYPE	rs7676614	0
GYPE	rs924234	0
GYPE	rs7681914	0
GYPE	rs2019159	0.00016143
GYPE	rs1375989	0
GYPE	rs1450250	0
GYPE	rs1375984	0
GYPE	rs1006406	0
GYPE	rs13105102	0
GYPE	rs4835305	0
GYPE	rs1822841	0
GYPE	rs9799404	0
GYPE	rs4287958	0
MGP	rs1861698	0.04691384
MGP	rs767842	0.03711097
MGP	rs6488724	0.04818842
MGP	rs4236	0.04818842
MGP	rs918121	0.00356692
MGP	rs10772817	0.00356692
MGP	rs10846082	0.00356692
MGP	rs10047541	0.00656304
MGP	rs2193360	0.00356692
MGP	rs2445370	0.00286541
MGP	rs4764137	0.01839552

Table 4: List of 353 significant gene-SNP associations in CAMP connected to IL1B

MGP	rs4764140	0.01839552
MGP	rs12320865	0.01839552
MGP	rs10772821	0.01839552
MESP1	rs4932231	9.98E-05
MESP1	rs10775247	0.00062155
MESP1	rs11629584	0.03970577
MESP1	rs2350479	0.04169588
MESP1	rs12900185	0.0009133
MESP1	rs8179043	0.0029768
MESP1	rs2289487	0.01755134
MESP1	rs3743115	0.00019305
