# Supplementary material for: A graphical model approach for inferring large-scale networks integrating gene expression and genetic polymorphism
Source: BMC Syst Biol. 2009 May 27;3:55. doi: 10.1186/1752-0509-3-55 (PMC2694152; doi:10.1186/1752-0509-3-55)
Supplement: Additional file 5 — List of 133 significant gene-SNP associations in GeneVar connected to IL1B. This table includes 133 significantly associated gene-SNP pairs where the gene is connected to IL1B in the gene-gene network in GeneVar dataset, including 25 SNPs which are associated with IL1B in the univariate analysis. [file 1752-0509-3-55-S5.pdf]

Table 5: List of 133 significant gene-SNP associations in GeneVar connected to IL1B

Gene	Marker	FDR adjusted	FDR adjusted p-value(univariate)
POMZP3	rs2527936	0	0.03533231
POMZP3	rs1799119	1.24E-06	0.01296288
RARRES2	rs2531003	0.01867692	0.10469867
RARRES2	rs7781827	0.01867692	0.10469867
RARRES2	rs17837498	0.01867692	0.10469867
RARRES2	rs7806429	0.01867692	0.10469867
RARRES2	rs7788316	0.01867692	0.10469867
RARRES2	rs2159236	0.03923293	0.02721225
RARRES2	rs2108854	0.01887046	0.04426168
RARRES2	rs11767726	0.01887046	0.04426168
RARRES2	rs2108852	0.01887046	0.04426168
RARRES2	rs1983440	0.02396519	0.04525796
RARRES2	rs4554381	0.01867692	0.03765169
RARRES2	rs10247016	0.03312511	0.33510482
RARRES2	rs11769348	0.02719584	0.07723716
RARRES2	rs3735172	0.02396519	0.04525796
RARRES2	rs3735171	0.02208012	0.05561203
RARRES2	rs3735170	0.04605039	0.03431515
RARRES2	rs3735169	0.02719584	0.08527999
RARRES2	rs17173617	0.0241618	0.02894063
RARRES2	rs3735167	0.02772438	0.04296529
RARRES2	rs10282458	0.02715603	0.01778385
RARRES2	rs17173681	0.02715282	0.02484121
RARRES2	rs17173682	0.02715282	0.02484121
RARRES2	rs7800196	0.02715282	0.02484121
PEX6	rs2235831	0.01867692	0.86165642
PEX6	rs6901007	2.68E-05	0.22778704
PEX6	rs2234185	3.75E-09	0.2606731
PEX6	rs4711731	4.94E-07	0.15855744
PEX6	rs4714634	2.40E-11	0.88546687
PEX6	rs3763236	0	0.77542493
PEX6	rs1053538	4.23E-13	0.75701222
PEX6	rs9471969	0.0383011	0.93767376
PEX6	rs9462853	0.00366831	0.50965271
PEX6	rs6927188	0.03472666	0.8613946
PEX6	rs9471970	0.03106452	0.49768082
PEX6	rs7744454	0.00895414	0.62232614
PEX6	rs6941212	0	0.2676202
PEX6	rs9471976	0	0.51970151
PEX6	rs13215983	0	0.4656736
PEX6	rs9462856	0	0.51970151
PEX6	rs11752813	0	0.25720507
PEX6	rs2296805	0	0.52233684
PEX6	rs2296804	0	0.57009701
PEX6	rs1129187	0	0.25720507
PEX6	rs2274517	0	0.51970151
PEX6	rs2274514	0	0.52252402
PEX6	rs3818554	0	0.58215754

Table 5: List of 133 significant gene-SNP associations in GeneVar connected to IL1B

PEX6	rs3805952	0	0.25720507
PEX6	rs3805951	0	0.19666036
PEX6	rs3293	0	0.62601901
PEX6	rs2395943	0	0.56719273
PEX6	rs9986447	0	0.27417893
PEX6	rs9462857	0	0.16073955
PEX6	rs9462859	0	0.25720507
PEX6	rs3805946	6.54E-14	0.32294169
GSTT1	rs140245	0	0.25540498
GSTT1	rs5760147	0	0.22615997
GSTT1	rs140289	0.00327083	0.42423456
GSTT1	rs6004011	0.01698887	0.02087259
GSTT1	rs412377	0.00558385	0.013559
GSTT1	rs8141342	0.01867692	0.01747013
GSTT1	rs6004014	0.01867692	0.01747013
GSTT1	rs5996650	0.01867692	0.01747013
GSTT1	rs5996651	0.01867692	0.01747013
GSTT1	rs7291786	0.01867692	0.01747013
GSTT1	rs140294	0.00196643	0.51995985
GSTT1	rs407257	0	0.22706327
GSTT1	rs8138555	0.01867692	0.01747013
GSTT1	rs5760176	0	0.24570395
GSTT1	rs17004811	0.00018376	0.55161876
GSTT1	rs738809	2.50E-07	0.47595873
PPIL3	rs3851973	5.05E-07	0.44564209
PPIL3	rs11892119	7.45E-07	0.48938063
PPIL3	rs7562391	5.05E-07	0.44564209
PPIL3	rs7606251	1.24E-13	0.49658077
PPIL3	rs2136600	1.24E-13	0.59623244
PPIL3	rs4035022	5.05E-07	0.5496953
PPIL3	rs6747253	1.01E-06	0.71746524
PPIL3	rs13384245	5.36E-09	0.41436921
PPIL3	rs10204787	5.36E-09	0.41436921
PPIL3	rs7588993	1.64E-11	0.67909524
PPIL3	rs6435066	7.85E-13	0.75403914
PPIL3	rs7917	7.85E-13	0.75403914
PPIL3	rs11894842	7.85E-13	0.75403914
PPIL3	rs4381763	5.36E-09	0.29367038
PPIL3	rs2307358	6.92E-09	0.46328207
DNASE1L3	rs7373012	0.00951778	0.43257197
DNASE1L3	rs7372278	0.01663003	0.45754462
DNASE1L3	rs4074657	0.0019174	0.44268833
DNASE1L3	rs7611872	0.02851448	0.31192302
DNASE1L3	rs7611951	0.02891578	0.17756752
DNASE1L3	rs9855113	0.01887046	0.36158936
DNASE1L3	rs4681818	0.01887046	0.36158936
DNASE1L3	rs3772985	0.00131683	0.03752063
DNASE1L3	rs4234389	0.00567764	0.23974309
DOK4	rs648742	0.00713976	0.79702626

Table 5: List of 133 significant gene-SNP associations in GeneVar connected to IL1B

DOK4	rs667380	0.04820041	0.46280225
DOK4	rs604965	6.36E-06	0.5395869
DOK4	rs636985	0.01675671	0.08814646
DOK4	rs8054916	0.00044522	0.63433544
SYNGR1	rs738331	0.03701712	0.96324225
SYNGR1	rs5757613	0.02619975	0.6292205
SYNGR1	rs137636	0.01212933	0.93609067
SYNGR1	rs5757617	0.02153096	0.84254966
SYNGR1	rs11704319	0.02153096	0.84254966
SYNGR1	rs12627761	0.02855795	0.74043163
SYNGR1	rs137685	0.00654778	0.63605678
SYNGR1	rs137687	0.00654778	0.63605678
SYNGR1	rs909685	3.68E-09	0.58012191
SYNGR1	rs2069235	3.68E-09	0.58012191
SYNGR1	rs715505	0.00024579	0.65747243
C21orf56	rs8126854	0.03078858	0.75448024
IRF5	rs4728142	1.59E-09	0.51216899
IRF5	rs752637	0	0.98420853
IRF5	rs3807306	0	0.67313353
IRF5	rs11761199	3.62E-12	0.38690749
IRF5	rs7808907	0	0.83099002
IRF5	rs1874328	0.00073026	0.41130397
IRF5	rs13242262	0	0.12015494
IRF5	rs10488630	0.01034952	0.79236558
IRF5	rs2280714	0	0.61970568
IRF5	rs10236569	0	0.51567697
IRF5	rs6966125	9.93E-08	0.91407344
IRF5	rs10229001	0	0.48296567
IRF5	rs2172876	5.03E-07	0.07602147
IRF5	rs4731534	0	0.54254611
IRF5	rs4731535	1.82E-07	0.07956415
IRF5	rs8043	1.82E-07	0.07956415
IRF5	rs1874332	1.82E-07	0.07956415
IRF5	rs2272347	0	0.42213294
IRF5	rs7789423	0	0.66942103
IRF5	rs6948928	0	0.66942103
